# AXIOME3: Automation, eXtension, and Integration Of Microbial Ecology

**DOI:** 10.1093/gigascience/giab006

**Published:** 2021-02-03

**Authors:** Daniel Min, Andrew C Doxey, Josh D Neufeld

**Affiliations:** Department of Biology, University of Waterloo, 200 University Avenue West, Waterloo, ONT N2L 3G1, Canada; Department of Biology, University of Waterloo, 200 University Avenue West, Waterloo, ONT N2L 3G1, Canada; Department of Biology, University of Waterloo, 200 University Avenue West, Waterloo, ONT N2L 3G1, Canada

**Keywords:** microbial ecology, 16S rRNA genes, SSU rRNA, QIIME2, interactive pipeline

## Abstract

**Background:**

Advances in high-throughput sequencing accessibility have democratized small subunit ribosomal RNA gene sequence data collection, coincident with an increasing availability of computational tools for sequence data processing, multivariate statistics, and data visualization. However, existing tools often require programming ability and frequent user intervention that may not be suitable for fast-paced and large-scale data analysis by end user microbiologists who are unfamiliar with the Linux command line environment or who prefer interactions with a GUI. Here we present AXIOME3, which is a completely redeveloped AXIOME pipeline that streamlines small subunit ribosomal RNA data analysis by managing QIIME2, R, and Python-associated analyses through an interactive web interface.

**Findings:**

AXIOME3 comes with web GUI to improve usability by simplifying configuration processes and task status tracking. Internally, it uses an automated pipeline that is wrapped around QIIME2 to generate a range of outputs including amplicon sequence variant tables, taxonomic classifications, phylogenetic trees, biodiversity metrics, and ordinations. The extension module for AXIOME3 provides advanced data visualization tools such as principal coordinate analysis, bubble plots, and triplot ordinations that can be used to visualize interactions between a distance matrix, amplicon sequence variant taxonomy, and sample metadata.

**Conclusions:**

Because repeat analysis of small subunit ribosomal RNA amplicon sequence data is challenging for those who have limited experience in command line environments, AXIOME3 now offers rapid and user-friendly options within an automated pipeline, with advanced data visualization tools and the ability for users to incorporate additional analyses easily through extension. AXIOME3 is completely open source (https://github.com/neufeld/AXIOME3, https://github.com/neufeld/AXIOME3-GUI), and researchers are encouraged to modify and redistribute the package.

## Findings

Advances in high-throughput DNA sequencing technologies have facilitated large-scale small subunit (SSU) ribosomal RNA (rRNA) data collection, which consequently increased the need for efficient computational tools. Although existing pipelines and databases such as QIIME2 [1], mothur [2], Ribosomal Database Project [3], and EzTaxon [4] provide modules to analyze amplicon data, they often require users to manually consolidate and execute individual workflow components. This may limit the efficiency of frequent repetitive analyses, especially for users who are inexperienced with the Linux terminal because several of the component tools must be performed in such an environment.

Previously, we developed the Automation, eXtension, and Integration Of Microbial Ecology (AXIOME) pipeline that enabled researchers to automate the analysis of SSU rRNA gene amplicon data easily [5], with most use cases involving management of the original QIIME [6] workflow. Coinciding with the release of QIIME2 [1], here we present AXIOME3, which is a completely redesigned version of AXIOME with greater emphases on usability, automation, and extension. AXIOME3 includes a web-based GUI to accommodate researchers who may be unfamiliar with traditional command line tools that are designed for the Linux environment [7]. Also, AXIOME3 provides an interactive pipeline that generates necessary data files and visual displays with minimal user intervention. In addition to enhanced usability and automation, AXIOME3 offers data visualization tools that are unique to this platform while also allowing extension to include other analyses, visualizations, and techniques to be integrated seamlessly.

### Usability

The AXIOME3 web GUI was designed to accommodate researchers who are unfamiliar with the Linux operating system environment, eliminating a potentially steep learning curve associated with traditional bioinformatics tools. Users can easily configure various options and start the automated analysis pipeline via a straightforward web interface. All usage-related information is embedded in the web interface so that users can avoid navigating to different resources in search of relevant information. Because a typical SSU rRNA gene amplicon data analysis may take several hours for a relatively large sample size, AXIOME3 assigns a unique session identifier to each analysis, which can be monitored and reloaded at any time. Users may optionally receive email notifications upon task queueing and completion.

AXIOME3 resolves potential installation conflicts by containerizing its software and operating system dependencies using Docker [8] and Docker Compose. Consequently, the only software requirements for AXIOME3 are Docker and Docker Compose, upon which the pipeline relies to have a consistent build environment. The web user interface is fully compatible with Chrome, Firefox, and Edge browsers.

### Automation

**Figure 1:**
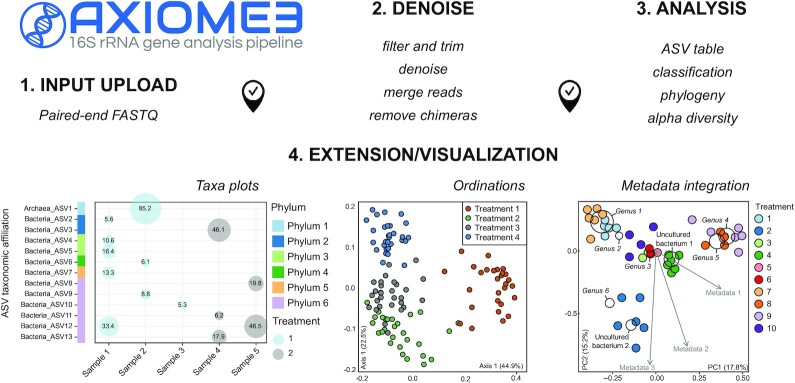
Schematic representation of the AXIOME3 pipeline workflow.

The AXIOME3 pipeline makes computational microbial research accessible to researchers who are unfamiliar with the Linux command line environment by automating common microbial research workflows. The core functionality of the pipeline relies on the QIIME2 package [1] and uses additional custom scripts to export QIIME2-formatted outputs to web-friendly formats (Fig. 1). Currently, it uses QIIME2 and its associated plugins to automatically process demultiplexed paired-end FASTQ reads. The AXIOME3 pipeline uses the DADA2 [9] plugin to denoise, dereplicate, remove chimeric reads, assemble sequences, and generate an amplicon sequence variant (ASV) table. The pipeline also supports batch analysis of samples from different sequencing runs. It performs denoising and assembly on each run for the samples belonging to the same run, and later combines individual ASV tables into a single merged ASV table. The AXIOME3 pipeline then assigns taxonomy to each ASV using the classifier that is trained on either the Ribosomal Database Project [3] or SILVA [10] database. Users have options to use the default classifier that comes with AXIOME3 or custom-trained classifiers. The pipeline then constructs a phylogenetic tree, calculates α-diversity indexes, β-diversity metrics, and ordination plots. Another key feature of the AXIOME3 pipeline is workflow checkpointing so that researchers can repeat only necessary analysis steps when small adjustments are made to the workflow. Furthermore, the AXIOME3 pipeline adheres to the extension and integration philosophy of the previous AXIOME package [5] so that any QIIME2 plugins, custom scripts, and novel analysis tools are integrated within the pipeline as a part of the automated workflow. Full extensibility and integrability are intended to encourage research community involvement, as well as ensure state-of-the-art microbial ecology research workflow.

### Extension

The extension module is unique to AXIOME3, with custom Python and R scripts used to visualize outputs of the interactive pipeline (Fig. 1). Currently, AXIOME3 supports three primary data reduction and visualization techniques: (i) taxonomy bubble plots, representing abundances of ASVs (or their associated taxonomic ranks) for samples; (ii) principal coordinate analysis, an ordination technique to project samples into multidimensional space while maximally preserving the original dissimilarity relationship between the samples [11]; and (iii) metadata integration using “triplots,” which simultaneously projects samples, taxonomic contributions to the samples as weighted averages, and the correlation between environmental factors and the samples as vectors within ordination space [12] (Fig. 1). All data visualization tools in the extension module are tailored to meet each researcher's specific needs. Various plot-specific elements (e.g., ordination axis selection) and general plot aesthetics (e.g., point colour, point size, font size, and plot size) are readily customizable. The extension module also supports visualization result previewing and downloading in raster and vector image file formats (e.g., PNG and PDF) so that users can iteratively explore and visualize their data with different customizations prior to downloading the final output files.

Additional data exploration, visualization, and statistical tools will be added in future releases of AXIOME3, and users are invited to participate in the development process.

### Workflow

A typical AXIOME3 workflow involves four modular analyses: Input Upload, Denoise, Analysis, and Extension/Visualization (Fig. 1). For the Input Upload module, users prepare manifest files listing absolute paths to input FASTQ files. Currently, AXIOME3 only supports demultiplexed paired-end FASTQ files as inputs. The output of this module is a summary of the input sequences in QIIME2 visualization format (.qzv), which could be used to determine the regions with low quality scores. Users denoise input sequences with optional removal of low-quality regions to improve the assembly result and generate the ASV table in the Denoise module. Subsequently, users generate the remaining outputs in the Analysis module using the generated ASV table. Each module acts as a checkpoint to the next module, which allows users to readily repeat part of the workflow upon failure or reconfiguration without rerunning the previous module. Optionally, data visualization tools in the Extension/Visualization module may be used to explore and visualize the ASV table or the ordination data.

### Comparison to related work

#### AXIOME

AXIOME3 offers several improvements compared to its predecessor AXIOME [5] and AXIOME2 (unpublished), even though it still abides by an extension and integration philosophy so that novel analysis tools and techniques can seamlessly be added to the workflow by collaborators. Because AXIOME3 is a wrapper around QIIME2 [1], it is capable of generating ASV-based features as opposed to operational taxonomic unit features generated by AXIOME, which used the original QIIME [6] package. Also, AXIOME3 renders a more visually appealing and accessible GUI because it leverages modern web browser technologies compared to AXIOME's Linux terminal–based GUI. AXIOME3 also aims to reduce the installation burden for end users by containerizing all its dependencies using Docker [8]. On the other hand, AXIOME requires users to manually install underlying packages and resolve potential dependency problems.

#### QIIME2 Studio

Although QIIME2 Studio provides a user-friendly GUI for amplicon sequence analysis, it has several limitations that are addressed by AXIOME3. First, QIIME2 data visualization files (.qzv) require the QIIME2 viewer, which adds an extra layer of complexity for end user microbiologists. Instead, AXIOME3 implements analysis and visualization within the same front-end interface, which simplifies the process for users. Second, QIIME2 Studio requires that users manually assemble individual workflow components. This limitation is addressed by AXIOME3 by enabling an automated workflow in which individual components are chained together into a single pipeline. This increased automation benefits users by simplifying repetitive workflows. The AXIOME3 pipeline also enables iterative data visualization and analysis, which allows users to easily interact with their data while customizing data visualizations. Importantly, AXIOME3 is not intended as a replacement for QIIME2 and QIIME2 Studio but is rather an automation tool that manages and extends QIIME2 capabilities.

#### Availability of AXIOME3

AXIOME3 is an actively maintained and developed open-source project and is available from GitHub (https://github.com/neufeld/AXIOME3; https://github.com/neufeld/AXIOME3-GUI). Note that only the AXIOME3 GUI (https://github.com/neufeld/AXIOME3-GUI) needs to be installed for end users and doing so will automatically install the pipeline as well. The AXIOME3 pipeline repository (https://github.com/neufeld/AXIOME3) is exclusively intended for developers and collaborators. AXIOME3 is cross-platform compatible and the web GUI currently supports Chrome, Firefox, and Edge. The only other software requirement for AXIOME3 is Docker and Docker Compose, which is required to ensure a consistent build environment. A tutorial and sample dataset, as well as instructions about collaboration, are included in the AXIOME3 GUI project home page.

## Availability of Supporting Source Code and Requirements

Project name: AXIOME3

Project home page: https://github.com/neufeld/AXIOME3 (AXIOME3 pipeline), https://github.com/neufeld/AXIOME3-GUI (AXIOME3 GUI)

Operating system(s): Platform independent

Browser support: Chrome, Firefox, Edge (AXIOME3 GUI)

Programming language: Python, Javascript

Other requirements: Docker (1.13.0+), Docker Compose (Version 3+)

License: BSD 3-Clause

Any restrictions to use by non-academics: No

## Data Availability

Snapshots of our code and other data further supporting this work are openly available in the GigaScience repository, GigaDB [13].

## Abbreviations

ASV: amplicon sequence variant; GUI: graphical user interface; rRNA: ribosomal RNA; SSU: small subunit.

## Competing Interests

The authors declare that they have no competing interests.

## Funding

In addition to Discovery grants to A.C.D. and J.D.N. from the Natural Sciences and Engineering Research Council of Canada (NSERC), this research was supported by an Ontario Research Fund: Research Excellence (ORF-RE) grant and a Collaborative Research and Development (CRD) grant from NSERC, both in partnership with the Nuclear Waste Management Organization (NWMO).

## Authors' Contributions

D.M. designed and implemented AXIOME3 and prepared the manuscript. A.C.D. and J.D.N. contributed to design and coordination of AXIOME3 and manuscript preparation. All authors read and approved the final manuscript.

## Supplementary Material

giab006_GIGA-D-20-00273_Original_Submission

giab006_GIGA-D-20-00273_Revision_1

giab006_Response_to_Reviewer_Comments_Original_Submission

giab006_Reviewer_1_Report_Original_Submission Julien Tremblay -- 10/14/2020 Reviewed

giab006_Reviewer_1_Report_Revision_1 Julien Tremblay -- 2/22/2020 Reviewed

giab006_Reviewer_2_Report_Original_Submission Todd Treangen -- 10/20/2020 Reviewed

giab006_Reviewer_2_Report_Revision_1 Todd Treangen -- 12/9/2020 Reviewed
