# Supplementary material for: AXIOME3: Automation, eXtension, and Integration Of Microbial Ecology
Source: Gigascience. 2021 Feb 3;10(2):giab006. doi: 10.1093/gigascience/giab006 (PMC7931817; doi:10.1093/gigascience/giab006)
Supplement: giab006_GIGA-D-20-00273_Original_Submission [file giab006_giga-d-20-00273_original_submission.pdf]

GigaScience

AXIOME3: Automation, eXtension, and Integration Of Microbial Ecology

--Manuscript Draft--

**Manuscript Number:**	GIGA-D-20-00273
**Full Title:**	AXIOME3: Automation, eXtension, and Integration Of Microbial Ecology
**Article Type:**	Technical Note
**Funding Information:**	Canadian Network for Research and Innovation in Machining Technology, Natural Sciences and Engineering Research Council of Canada — Dr. Josh Neufeld
**Abstract:**	Background<br><br>Advances in high-throughput sequencing accessibility have democratized small subunit rRNA gene sequence data collection, coincident with the increasing availability of computational tools for sequence data processing, multivariate statistics, and data visualization. However, existing tools often require command line familiarity, programming ability, and frequent user intervention that may not be suitable for fast-paced and large-scale data analysis by a wide range of end user microbiologists. Here we present AXIOME3, which is a completely redeveloped AXIOME pipeline that streamlines SSU rRNA data analysis by managing QIIME2, R, and Python-associated analyses through an interactive web interface.<br><br>Findings<br><br>The AXIOME3 pipeline comes with web graphical user interface to improve usability by simplifying configuration processes and task status tracking. Internally, it uses an automated pipeline that is wrapped around QIIME2 to generate a range of outputs including amplicon sequence variant tables, taxonomic classifications, phylogenetic trees, biodiversity metrics, and ordinations. The extension module for AXIOME3 provides advanced data visualization tools such as principle coordinate analysis, bubble plots, and triplot ordinations that can be used to visualize interactions between a distance matrix, ASV taxonomy, and sample metadata.<br><br>Conclusions<br><br>Because mass collection of SSU rRNA gene amplicon sequence data presents a challenge to efficiently analyze the large-scale data in a timely manner, AXIOME3 now offers rapid and user-friendly options within an automated pipeline, with advanced data visualization tools and the ability for users to incorporate additional analyses easily through extension. The AXIOME3 pipeline is completely open source (https://github.com/neufeld/AXIOME3, https://github.com/neufeld/AXIOME3-GUI) and researchers are encouraged to modify and redistribute the package.
**Corresponding Author:**	Josh Neufeld<br>University of Waterloo<br>Waterloo, CANADA
**Corresponding Author Secondary Information:**	
**Corresponding Author's Institution:**	University of Waterloo
**Corresponding Author's Secondary Institution:**	
**First Author:**	Daniel Min
**First Author Secondary Information:**	
**Order of Authors:**	Daniel Min
	Andrew C Doxey

Powered by Editorial Manager® and ProduXion Manager® from Aries Systems Corporation

	Josh Neufeld
**Order of Authors Secondary Information:**	
**Additional Information:**	
**Question**	**Response**
Are you submitting this manuscript to a special series or article collection?	No
**Experimental design and statistics**<br><br>Full details of the experimental design and statistical methods used should be given in the Methods section, as detailed in our Minimum Standards Reporting Checklist. Information essential to interpreting the data presented should be made available in the figure legends.<br><br>Have you included all the information requested in your manuscript?	Yes
**Resources**<br><br>A description of all resources used, including antibodies, cell lines, animals and software tools, with enough information to allow them to be uniquely identified, should be included in the Methods section. Authors are strongly encouraged to cite Research Resource Identifiers (RRIDs) for antibodies, model organisms and tools, where possible.<br><br>Have you included the information requested as detailed in our Minimum Standards Reporting Checklist?	Yes
**Availability of data and materials**<br><br>All datasets and code on which the conclusions of the paper rely must be either included in your submission or deposited in publicly available repositories (where available and ethically appropriate), referencing such data using a unique identifier in the references and in	Yes

the "Availability of Data and Materials" section of your manuscript.<br><br>Have you have met the above requirement as detailed in our Minimum Standards Reporting Checklist?	

Click here to access/download;Manuscript;Minms_final.docx

AXIOME3: Automation, eXtension, and Integration Of Microbial Ecology

Min D, Doxey AC, Neufeld JD*

University of Waterloo, 200 University Avenue West, Waterloo, Ontario, Canada, N2L 3G1

*Correspondence to: jneufeld@uwaterloo.ca

Abstract

Background

Advances in high-throughput sequencing accessibility have democratized small subunit rRNA gene sequence data collection, coincident with the increasing availability of computational tools for sequence data processing, multivariate statistics, and data visualization. However, existing tools often require command line familiarity, programming ability, and frequent user intervention that may not be suitable for fast-paced and large-scale data analysis by a wide range of end user microbiologists. Here we present AXIOME3, which is a completely redeveloped AXIOME pipeline that streamlines SSU rRNA data analysis by managing QIIME2, R, and Python-associated analyses through an interactive web interface.

Findings

The AXIOME3 pipeline comes with web graphical user interface to improve usability by simplifying configuration processes and task status tracking. Internally, it uses an automated pipeline that is wrapped around QIIME2 to generate a range of outputs including amplicon

sequence variant tables, taxonomic classifications, phylogenetic trees, biodiversity metrics, and ordinations. The extension module for AXIOME3 provides advanced data visualization tools such as principle coordinate analysis, bubble plots, and triplot ordinations that can be used to visualize interactions between a distance matrix, ASV taxonomy, and sample metadata.

Conclusions

Because mass collection of SSU rRNA gene amplicon sequence data presents a challenge to efficiently analyze the large-scale data in a timely manner, AXIOME3 now offers rapid and user-friendly options within an automated pipeline, with advanced data visualization tools and the ability for users to incorporate additional analyses easily through extension. The AXIOME3 pipeline is completely open source (https://github.com/neufeld/AXIOME3, https://github.com/neufeld/AXIOME3-GUI) and researchers are encouraged to modify and redistribute the package.

Keywords

Microbial ecology, 16S rRNA genes, SSU rRNA, QIIME2, interactive pipeline

Findings

Advances in high-throughput DNA sequencing technologies have facilitated large-scale small subunit (SSU) ribosomal RNA (rRNA) data collection, which consequently increased the need for efficient computational tools. Although existing pipelines and databases such as QIIME2 [1], mothur [2], Ribosomal Database Project (RDP) [3], and EzTaxon [4] provide modules to analyze amplicon data, they often require users to manually consolidate and execute individual workflow components, which may limit the efficiency of frequent repetitive analyses.

Previously we developed the Automation, eXtension, and Integration Of Microbial Ecology (AXIOME) pipeline that enabled researchers to automate the analysis of SSU rRNA gene amplicon data with ease [5], with most use cases involving management of the original QIIME [6] workflow. Coinciding with the release of QIIME2 [1], here we present AXIOME3, which is a completely redesigned version of AXIOME with greater emphases on usability, automation, and extension. The AXIOME3 pipeline includes a web-based graphical user interface (GUI) to accommodate researchers who may be unfamiliar with traditional command line tools that are designed for the Linux environment [7]. Also, AXIOME3 provides an interactive pipeline that generates necessary data files and displays with minimal user intervention. In addition to enhanced usability and automation, AXIOME3 offers advanced data visualization tools that are not available in the currently existing tools and allows other analysis and visualization tools and techniques to be integrated seamlessly.

Usability

The AXIOME3 web GUI was designed to accommodate researchers who may be unfamiliar with the Linux operating system environment, eliminating a potentially steep learning curve associated with traditional bioinformatics tools. Users can easily configure various options and start the automated analysis pipeline via a straightforward web interface. All usage-related information is embedded in the web interface so that users can avoid navigating to different resources in search of relevant information. Because a typical SSU rRNA gene amplicon data analysis may take several hours for a relatively large sample size, AXIOME3 assigns a unique session identifier to each analysis, which can be monitored and re-loaded at any time. Users may optionally receive email notifications upon task queueing and completion.

The AXIOME3 pipeline resolves potential installation conflicts by containerizing its software and operating system dependencies using Docker [8] and Docker Compose. Consequently, the only software requirements for AXIOME3 is Docker and Docker Compose, upon which the pipeline relies to have a consistent build environment. The web user interface is fully compatible with Chrome, Firefox, and Edge browsers.

Automation

The AXIOME3 pipeline accelerates research by automating common workflows employed by microbial ecologists. The core functionality of the pipeline relies on the QIIME2 package [1] and uses additional custom scripts to export QIIME2-formatted outputs to web-friendly formats (Figure 1). Currently, it uses a series of QIIME2 and its associated plugins to automatically process demultiplexed paired-end FASTQ reads. The AXIOME3 pipeline uses the

DADA2 [9] plugin to denoise, dereplicate, remove chimeric reads, assemble sequences, and generate amplicon sequence variant (ASV) table. The pipeline also supports batch analysis of samples from different sequencing runs. It performs denoising and assembly on each run for the samples belonging to the same run, and later combines the individual ASV tables into a single merged ASV table. The AXIOME3 pipeline then assigns taxonomy to each ASV using the classifier that is trained on the SILVA database [10]. Users have options to use the default classifier that comes with AXIOME3 or custom-trained classifiers. The pipeline then constructs a phylogenetic tree, calculates alpha diversity indexes, beta diversity metrics, and ordination plots. Because several amplicon data analysis tasks are time consuming and resource heavy, another key feature of AXIOME3 is workflow checkpointing so that researchers can repeat only necessary analysis steps when small adjustments are made to the workflow. Furthermore, the AXIOME3 pipeline adheres to the extension and integration philosophy of the previous AXIOME package [5] so that any QIIME2 plugins, custom scripts, and novel analysis tools are integrated within the pipeline as a part of the automated workflow. Full extensibility and integrability are intended to encourage research community involvement as well as to ensure state of the art microbial ecology research workflow.

Extension

The extension module is unique to AXIOME3, with custom Python and R scripts used to visualize outputs of the interactive pipeline (Figure 1). Currently, AXIOME3 supports three primary data dimensionality reduction and visualization techniques: (1) taxonomy bubble plots, representing abundances of ASVs (or their associated taxonomic ranks) for samples, (2) principal coordinate analysis (PCoA), an ordination technique to project samples into

multidimensional space while maximally preserving the original dissimilarity relationship between the samples [11], and (3) metadata integration using “triplots”, which simultaneously project samples, taxonomic contributions to the samples as weighted averages, and the correlation between environmental factors and the samples as vectors within ordination space [12] (Figure 1). All data visualization tools in the extension module are tailored to meet each researcher’s specific needs. Various plot-specific elements (e.g., ordination axis selection) and general plot aesthetics (e.g., point colour, point size, font size, and plot size) are readily customizable. The extension module also supports visualization result previewing and downloading in raster and vector image file formats (e.g., PNG and PDF) so that users can iteratively explore and visualize their data with different customizations and download the final output files.

Additional data exploration, visualization, and statistical tools will be added in future releases of AXIOME3 and users are invited to participate in the development process.

Workflow

A typical AXIOME3 workflow involves four modular analyses: Input Upload, Denoise, Analysis, and Extension/Visualization (Figure 1). In the Input Upload module, users prepare manifest files listing absolute paths to input FASTQ files. Currently, AXIOME3 only supports demultiplexed paired-end FASTQ files as inputs. The output of this module is a summary of the input sequences in QIIME2 visualization format (.qzv) which could be used to determine the regions with low quality scores. Then, users denoise the input sequences with optional removal of low-quality regions to improve the assembly result and generate the ASV table in the Denoise

module. Users then generate the rest of the outputs in the Analysis module using the generated ASV table. Each module acts as a checkpoint to the next module, which allows users to readily repeat part of the workflow upon failure or reconfiguration without re-running the previous module. Optionally, data visualization tools in the Extension/Visualization module may be used to explore and visualize ASV table or the ordination data.

Comparison to related work

Although QIIME2 Studio provides a user-friendly graphical user interface for amplicon sequencing analysis, it has several limitations that are addressed by AXIOME3. First, QIIME2 data visualization files (.qzv) requires the QIIME2 viewer, which adds an extra layer of complexity to end user biologists. Instead, AXIOME3 implements analysis and visualization within the same front-end interface, which simplifies the process for users. Second, QIIME2 Studio requires that users manually assemble individual workflow components. This limitation is addressed by AXIOME3 by enabling an automated workflow in which individual components are chained together into a single pipeline. This increased automation benefits users by simplifying repetitive workflows. The AXIOME3 pipeline also enables iterative data visualization and analysis, which allows users to easily interact with their data while customizing and optimizing data visualizations. Importantly, AXIOME3 is not intended as a replacement of QIIME2 and QIIME2 Studio, but is rather an automation tool that extends QIIME2 capabilities.

Availability of AXIOME3

AXIOME3 is an actively maintained and developed open-source project, and is available from GitHub (https://github.com/neufeld/AXIOME3; https://github.com/neufeld/AXIOME3-GUI). AXIOME3 is cross-platform compatible and the web GUI currently supports Chrome, Firefox, and Edge. The only other software requirement for AXIOME3 is Docker and Docker Compose on which it depends to have a consistent build environment.

Availability and Requirements

Project name: AXIOME3

Project home page: https://github.com/neufeld/AXIOME3 (AXIOME3 pipeline), https://github.com/neufeld/AXIOME3-GUI (AXIOME3 web GUI)

Operating system(s): Platform independent

Browser support: Chrome, Firefox, Edge (AXIOME3 web GUI)

Programming language: Python, Javascript

Other requirements: Docker (1.13.0+), Docker Compose (Version 3+)

License: BSD 3-Clause

Any restrictions to use by non-academics: No

Abbreviations

SSU: small subunit; rRNA: ribosomal RNA; ASV: amplicon sequence variant; PCoA: principal coordinate analysis

Competing Interests

The authors declare no competing interests

Authors' contributions

DM designed and implemented AXIOME3 and prepared manuscript. ACD contributed to design and coordination of AXIOME3 and manuscript preparation. JDN contributed to design and coordination of AXIOME3 and manuscript preparation. All authors read and approved the final manuscript.

Acknowledgements

In addition to Discovery grants to ACD and JDN from the Natural Sciences and Engineering Research Council of Canada (NSERC), this research was supported by an Ontario Research Fund: Research Excellence (ORF-RE) grant and a Collaborative Research and Development (CRD) grant from NSERC, both in partnership with the Nuclear Waste Management Organization (NWMO).

References

[1] Bolyen E, Rideout JR, Dillon MR, Bokulich NA, Abnet CC, Al-Ghalith GA, et al. **Reproducible, interactive, scalable and extensible microbiome data science using QIIME 2**. *Nat. Biotechnol*. 2019; **37**:852–857.

[2] Schloss PD, Westcott SL, Ryabin T, Hall JR, Hartmann M, Hollister EB, et al. **Introducing mothur: Open-source, platform-independent, community-supported software for describing and comparing microbial communities.** *Appl Environ Microbiol*. 2009; **75:**7537-7541.

[3] Cole JR, Wang Q, Fish JA, Chai B, McGarrell DM, Sun Y, et al. **Ribosomal Database Project: Data and tools for high throughput rRNA analysis.** *Nucleic Acids Res*. 2014; **42:**D633–D642.

[4] Kim OS, Cho YJ, Lee K, Yoon SH, Kim M, Na H, et al. **Introducing EzTaxon-e: A prokaryotic 16S rRNA gene sequence database with phylotypes that represent uncultured species.** *Int J Syst Evol Microbiol*. 2012; **62:**716-721.

[5] Lynch MDJ, Masella AP, Hall MW, Bartram AK, Neufeld JD. **AXIOME: Automated exploration of microbial diversity.** *Gigascience*. 2013; **2:**3.

[6] Caporaso JG, Kuczynski J, Stombaugh J, Bittinger K, Bushman FD, Costello EK, et al. **QIIME allows analysis of high-throughput community sequencing data.** *Nat. Methods*. 2010; **7:**335–336.

[7] Carvalho BS, Rustici G. **The challenges of delivering bioinformatics training in the analysis of high-throughput data.** *Brief Bioinform*. 2013; **14:**538-547.

[8] Merkel D. **Docker: lightweight Linux containers for consistent development and deployment.** *Linux J*. 2014; **2014:**2.

[9] Callahan BJ, McMurdie PJ, Rosen MJ, Han AW, Johnson AJA, Holmes SP. DADA2: **High-resolution sample inference from Illumina amplicon data**. *Nat Methods*. 2016; **13:** 581–583.

[10] Quast C, Pruesse E, Yilmaz P, Gerken J, Schweer T, Yarza P, et al. **The SILVA ribosomal RNA gene database project: Improved data processing and web-based tools.** *Nucleic Acids Res*. 2013; **41:**D590-D596.

[11] Legendre P, Legendre L. **Chapter 9 - Ordination in reduced space.** In: Legendre P, Legendre L, editors. *Numer Ecol.* Amsterdam: Elsevier; 2012. p. 425–520.

[12] Borcard D, Gillet F, Legendre P, Borcard D, Gillet F, Legendre P. **Unconstrained Ordination.** In: *Numer Ecol with R. Use R*. Berlin: Springer; 2011. p. 115-151.

Figure legends

Figure 1. Schematic representation of the AXIOME3 pipeline workflow.

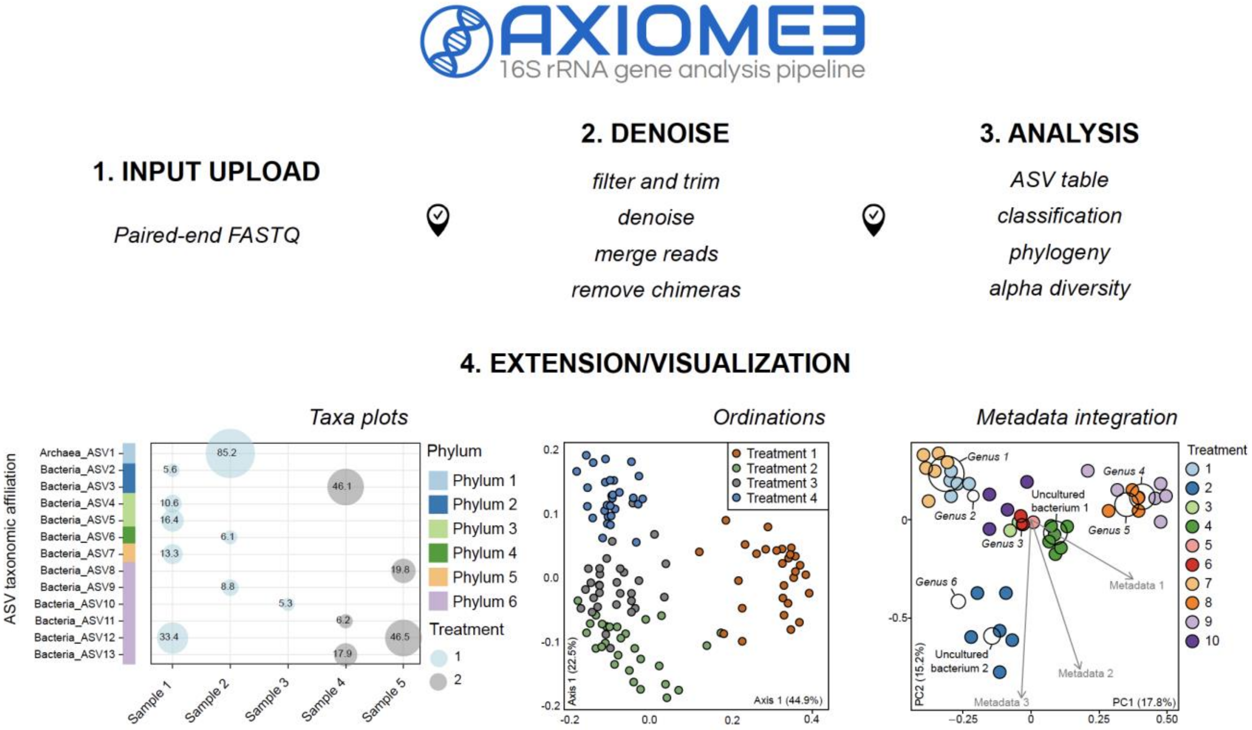

Click here to access/download;Figure;Figure 1.pdf

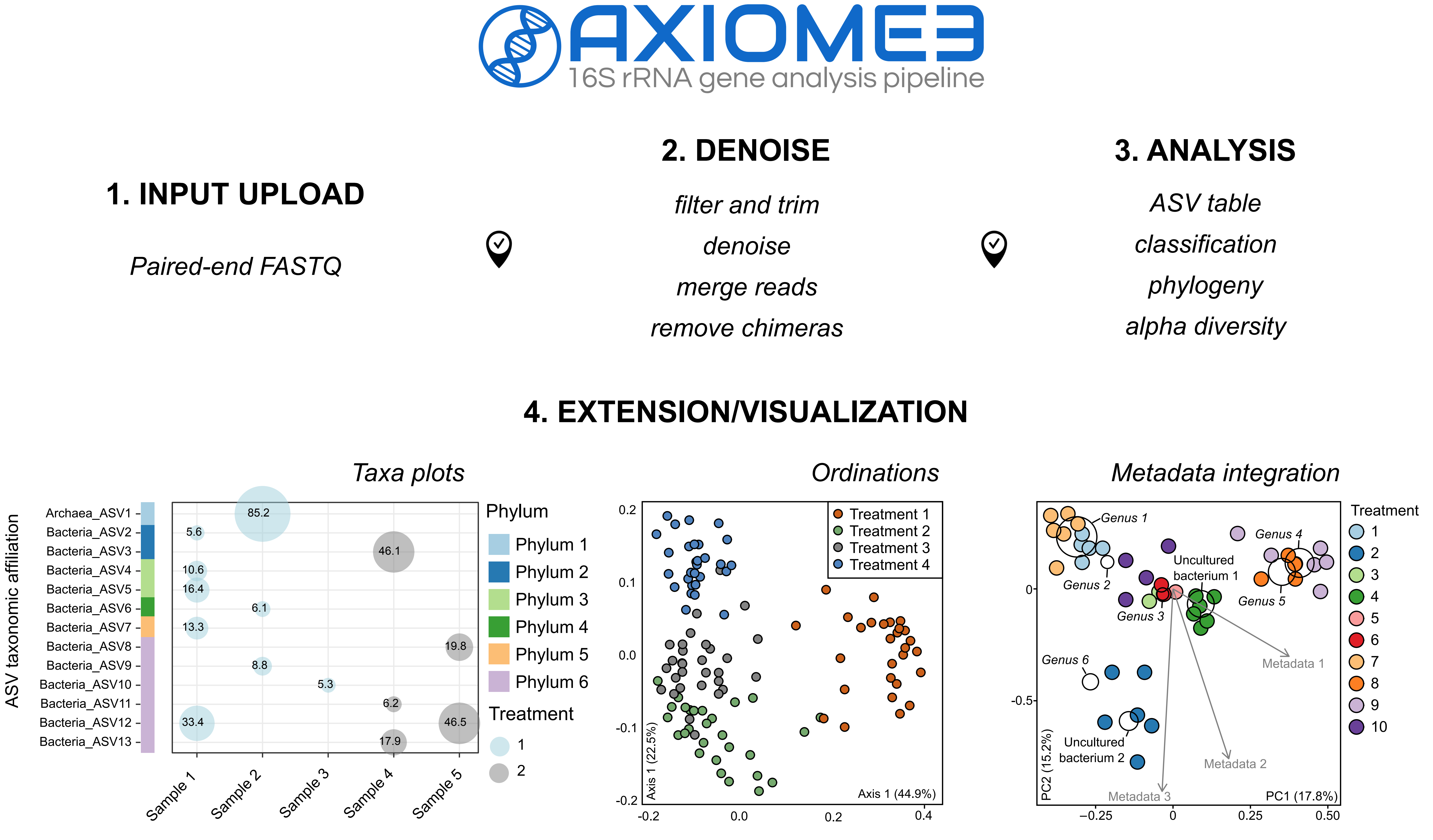

Click here to access/download;Personal Cover;Minms_coverletter.pdf

UNIVERSITY OF WATERLOO

DEPARTMENT OF BIOLOGY
519-888-4567 ext. 32569 | fax 519-746-0614
uwaterloo.ca/biology

GigaScience; September 7, 2020

Dear Editor,

We hereby submit a manuscript entitled “AXIOME3: Automation, eXtension, and Integration Of Microbial Ecology” for publication as an Technical Note in GigaScience. The software has not been submitted for publication elsewhere while under consideration for GigaScience. A single figure is included at the end of the upload file.

High-throughput sequencing of ribosomal RNA genes and the development of computational pipelines (e.g., QIIME, mothur) have transformed the study of microbial ecology. However, the high degree of user intervention required to analyze large sequence datasets and the limited analyses available through existing software packages present barriers to user-friendly management and customization of data workflows. Here we present AXIOME3, which is a user-friendly, open source and customizable workflow for bioinformatic analysis of 16S rRNA gene sequences. AXIOME3 simplifies and customizes analyses with other platforms and scripts, replacing AXIOME in the same way that QIIME2 replaced QIIME. AXIOME3 serves as a companion to any native QIIME2 install, has an associated GUI, and has been beta tested extensively by multiple users. Because AXIOME3 will be useful to microbial ecologists who analyze amplicon sequence data, we believe that this manuscript is ideally suited for publication as a Technical Note in GigaScience.

Thank you in advance for your consideration of this manuscript.

Sincerely,

Josh D. Neufeld; Professor

519-888-4567 | uwaterloo.ca | 200 UNIVERSITY AVENUE WEST, WATERLOO, ON, CANADA N2L 3G1
