# Supplementary material for: AXIOME3: Automation, eXtension, and Integration Of Microbial Ecology
Source: Gigascience. 2021 Feb 3;10(2):giab006. doi: 10.1093/gigascience/giab006 (PMC7931817; doi:10.1093/gigascience/giab006)
Supplement: giab006_GIGA-D-20-00273_Revision_1 [file giab006_giga-d-20-00273_revision_1.pdf]

GigaScience

AXIOME3: Automation, eXtension, and Integration Of Microbial Ecology

--Manuscript Draft--

**Manuscript Number:**	GIGA-D-20-00273R1
**Full Title:**	AXIOME3: Automation, eXtension, and Integration Of Microbial Ecology
**Article Type:**	Technical Note
**Funding Information:**	Canadian Network for Research and Innovation in Machining Technology, Natural Sciences and Engineering Research Council of Canada — Dr. Josh Neufeld
**Abstract:**	Background<br><br>Advances in high-throughput sequencing accessibility have democratized small subunit rRNA gene sequence data collection, coincident with the increasing availability of computational tools for sequence data processing, multivariate statistics, and data visualization. However, existing tools often require programming ability and frequent user intervention that may not be suitable for fast-paced and large-scale data analysis by end user microbiologists who are unfamiliar with the Linux command line environment or prefer interactions with a graphical user interface. Here we present AXIOME3, which is a completely redeveloped AXIOME pipeline that streamlines SSU rRNA data analysis by managing QIIME2, R, and Python-associated analyses through an interactive web interface.<br><br>Findings<br><br>AXIOME3 comes with web graphical user interface to improve usability by simplifying configuration processes and task status tracking. Internally, it uses an automated pipeline that is wrapped around QIIME2 to generate a range of outputs including amplicon sequence variant tables, taxonomic classifications, phylogenetic trees, biodiversity metrics, and ordinations. The extension module for AXIOME3 provides advanced data visualization tools such as principle coordinate analysis, bubble plots, and triplot ordinations that can be used to visualize interactions between a distance matrix, ASV taxonomy, and sample metadata.<br><br>Conclusions<br><br>Because repeat analysis of SSU rRNA amplicon sequence data is challenging for those who have limited experience in command line environments, AXIOME3 now offers rapid and user-friendly options within an automated pipeline, with advanced data visualization tools and the ability for users to incorporate additional analyses easily through extension. AXIOME3 is completely open source (https://github.com/neufeld/AXIOME3 , https://github.com/neufeld/AXIOME3-GUI) and researchers are encouraged to modify and redistribute the package.
**Corresponding Author:**	Josh Neufeld<br>University of Waterloo<br>Waterloo, CANADA
**Corresponding Author Secondary Information:**	
**Corresponding Author's Institution:**	University of Waterloo
**Corresponding Author's Secondary Institution:**	
**First Author:**	Daniel Min
**First Author Secondary Information:**	
**Order of Authors:**	Daniel Min<br>Andrew C Doxey

Powered by Editorial Manager® and ProduXion Manager® from Aries Systems Corporation

	Josh Neufeld
**Order of Authors Secondary Information:**	
**Response to Reviewers:**	See attached file (with italics used to distinguish reviewer comments)
**Additional Information:**	
**Question**	**Response**
Are you submitting this manuscript to a special series or article collection?	No
**Experimental design and statistics**<br><br>Full details of the experimental design and statistical methods used should be given in the Methods section, as detailed in our Minimum Standards Reporting Checklist. Information essential to interpreting the data presented should be made available in the figure legends.<br><br>Have you included all the information requested in your manuscript?	Yes
**Resources**<br><br>A description of all resources used, including antibodies, cell lines, animals and software tools, with enough information to allow them to be uniquely identified, should be included in the Methods section. Authors are strongly encouraged to cite Research Resource Identifiers (RRIDs) for antibodies, model organisms and tools, where possible.<br><br>Have you included the information requested as detailed in our Minimum Standards Reporting Checklist?	Yes
**Availability of data and materials**<br><br>All datasets and code on which the conclusions of the paper rely must be either included in your submission or deposited in publicly available repositories (where available and ethically appropriate), referencing such data using	Yes

a unique identifier in the references and in the “Availability of Data and Materials” section of your manuscript.<br><br>Have you have met the above requirement as detailed in our Minimum Standards Reporting Checklist?	

Manuscript revised with DOI

Click here to access/download;Manuscript;AXIOME3_revised w DOI.docx

AXIOME3: Automation, eXtension, and Integration Of Microbial Ecology

Min D, Doxey AC, Neufeld JD*

University of Waterloo, 200 University Avenue West, Waterloo, Ontario, Canada, N2L 3G1

*Correspondence to: jneufeld@uwaterloo.ca

Abstract

Background

Advances in high-throughput sequencing accessibility have democratized small subunit rRNA gene sequence data collection, coincident with the increasing availability of computational tools for sequence data processing, multivariate statistics, and data visualization. However, existing tools often require programming ability and frequent user intervention that may not be suitable for fast-paced and large-scale data analysis by end user microbiologists who are unfamiliar with the Linux command line environment or prefer interactions with a graphical user interface. Here we present AXIOME3, which is a completely redeveloped AXIOME pipeline that streamlines SSU rRNA data analysis by managing QIIME2, R, and Python-associated analyses through an interactive web interface.

Findings

AXIOME3 comes with web graphical user interface to improve usability by simplifying configuration processes and task status tracking. Internally, it uses an automated pipeline that is

wrapped around QIIME2 to generate a range of outputs including amplicon sequence variant tables, taxonomic classifications, phylogenetic trees, biodiversity metrics, and ordinations. The extension module for AXIOME3 provides advanced data visualization tools such as principle coordinate analysis, bubble plots, and triplot ordinations that can be used to visualize interactions between a distance matrix, ASV taxonomy, and sample metadata.

Conclusions

Because repeat analysis of SSU rRNA amplicon sequence data is challenging for those who have limited experience in command line environments, AXIOME3 now offers rapid and user-friendly options within an automated pipeline, with advanced data visualization tools and the ability for users to incorporate additional analyses easily through extension. AXIOME3 is completely open source (https://github.com/neufeld/AXIOME3, https://github.com/neufeld/AXIOME3-GUI) and researchers are encouraged to modify and redistribute the package.

Keywords

Microbial ecology, 16S rRNA genes, SSU rRNA, QIIME2, interactive pipeline

Findings

Advances in high-throughput DNA sequencing technologies have facilitated large-scale small subunit (SSU) ribosomal RNA (rRNA) data collection, which consequently increased the need for efficient computational tools. Although existing pipelines and databases such as QIIME2 [1], mothur [2], Ribosomal Database Project (RDP) [3], and EzTaxon [4] provide modules to analyze amplicon data, they often require users to manually consolidate and execute individual workflow components. This may limit the efficiency of frequent repetitive analyses, especially for users

who are inexperienced with the Linux terminal because several of the component tools must be performed in such an environment.

Previously, we developed the Automation, eXtension, and Integration Of Microbial Ecology (AXIOME) pipeline that enabled researchers to automate the analysis of SSU rRNA gene amplicon data with ease [5], with most use cases involving management of the original QIIME [6] workflow. Coinciding with the release of QIIME2 [1], here we present AXIOME3, which is a completely redesigned version of AXIOME with greater emphases on usability, automation, and extension. AXIOME3 includes a web-based graphical user interface (GUI) to accommodate researchers who may be unfamiliar with traditional command line tools that are designed for the Linux environment [7]. Also, AXIOME3 provides an interactive pipeline that generates necessary data files and visual displays with minimal user intervention. In addition to enhanced usability and automation, AXIOME3 offers advanced data visualization tools that are unique to this platform while also allowing extension to include other analyses, visualizations, and techniques to be integrated seamlessly.

Usability

The AXIOME3 web GUI was designed to accommodate researchers who are be unfamiliar with the Linux operating system environment, eliminating a potentially steep learning curve associated with traditional bioinformatics tools. Users can easily configure various options and start the automated analysis pipeline via a straightforward web interface. All usage-related information is embedded in the web interface so that users can avoid navigating to different resources in search of relevant information. Because a typical SSU rRNA gene amplicon data

analysis may take several hours for a relatively large sample size, AXIOME3 assigns a unique session identifier to each analysis, which can be monitored and re-loaded at any time. Users may optionally receive email notifications upon task queueing and completion.

AXIOME3 resolves potential installation conflicts by containerizing its software and operating system dependencies using Docker [8] and Docker Compose. Consequently, the only software requirements for AXIOME3 is Docker and Docker Compose, upon which the pipeline relies to have a consistent build environment. The web user interface is fully compatible with Chrome, Firefox, and Edge browsers.

Automation

The AXIOME3 pipeline enables computational microbial research accessible for researchers who are unfamiliar with the Linux command line environment by automating common microbial research workflows. The core functionality of the pipeline relies on the QIIME2 package [1] and uses additional custom scripts to export QIIME2-formatted outputs to web-friendly formats (Figure 1). Currently, it uses a series of QIIME2 and its associated plugins to automatically process demultiplexed paired-end FASTQ reads. The AXIOME3 pipeline uses the DADA2 [9] plugin to denoise, dereplicate, remove chimeric reads, assemble sequences, and generate an amplicon sequence variant (ASV) table. The pipeline also supports batch analysis of samples from different sequencing runs. It performs denoising and assembly on each run for the samples belonging to the same run, and later combines the individual ASV tables into a single merged ASV table. The AXIOME3 pipeline then assigns taxonomy to each ASV using the classifier that is trained on either the RDP [3] or SILVA [10] databases. Users have options to

use the default classifier that comes with AXIOME3 or custom-trained classifiers. The pipeline then constructs a phylogenetic tree, calculates alpha diversity indexes, beta diversity metrics, and ordination plots. Another key feature of the AXIOME3 pipeline is workflow checkpointing so that researchers can repeat only necessary analysis steps when small adjustments are made to the workflow. Furthermore, the AXIOME3 pipeline adheres to the extension and integration philosophy of the previous AXIOME package [5] so that any QIIME2 plugins, custom scripts, and novel analysis tools are integrated within the pipeline as a part of the automated workflow. Full extensibility and integrability are intended to encourage research community involvement as well as to ensure state of the art microbial ecology research workflow.

Extension

The extension module is unique to AXIOME3, with custom Python and R scripts used to visualize outputs of the interactive pipeline (Figure 1). Currently, AXIOME3 supports three primary data reduction and visualization techniques: (1) taxonomy bubble plots, representing abundances of ASVs (or their associated taxonomic ranks) for samples, (2) principal coordinate analysis (PCoA), an ordination technique to project samples into multidimensional space while maximally preserving the original dissimilarity relationship between the samples [11], and (3) metadata integration using “triplots”, which simultaneously project samples, taxonomic contributions to the samples as weighted averages, and the correlation between environmental factors and the samples as vectors within ordination space [12] (Figure 1). All data visualization tools in the extension module are tailored to meet each researcher’s specific needs. Various plot-specific elements (e.g., ordination axis selection) and general plot aesthetics (e.g., point colour, point size, font size, and plot size) are readily customizable. The extension module also supports

visualization result previewing and downloading in raster and vector image file formats (e.g., PNG and PDF) so that users can iteratively explore and visualize their data with different customizations prior to downloading the final output files.

Additional data exploration, visualization, and statistical tools will be added in future releases of AXIOME3 and users are invited to participate in the development process.

Workflow

A typical AXIOME3 workflow involves four modular analyses: Input Upload, Denoise, Analysis, and Extension/Visualization (Figure 1). For the Input Upload module, users prepare manifest files listing absolute paths to input FASTQ files. Currently, AXIOME3 only supports demultiplexed paired-end FASTQ files as inputs. The output of this module is a summary of the input sequences in QIIME2 visualization format (.qzv), which could be used to determine the regions with low quality scores. Users denoise input sequences with optional removal of low-quality regions to improve the assembly result and generate the ASV table in the Denoise module. Subsequently, users generate the remaining outputs in the Analysis module using the generated ASV table. Each module acts as a checkpoint to the next module, which allows users to readily repeat part of the workflow upon failure or reconfiguration without re-running the previous module. Optionally, data visualization tools in the Extension/Visualization module may be used to explore and visualize ASV table or the ordination data.

Comparison to related work

AXIOME

AXIOME3 offers several improvements compared to its predecessor AXIOME [5], and AXIOME2 (unpublished), even though it still abides by extension and integration philosophy so that novel analysis tools and techniques can seamlessly be added to the workflow by collaborators. Because AXIOME3 is a wrapper around QIIME2 [1], it is capable of generating ASV-based features as opposed to operational taxonomic unit (OTU) features generated by AXIOME, which used the original QIIME [6] package. Also, AXIOME3 renders more visually appealing and accessible GUI because it leverages modern web browser technologies compared to AXIOME's Linux terminal based GUI. AXIOME3 also aims to reduce installation burden for end users by containerizing all its dependencies using Docker [8]. On the other hand, AXIOME requires users to manually install underlying packages and resolve potential dependency problems.

QIIME2

Although QIIME2 Studio provides a user-friendly graphical user interface for amplicon sequencing analysis, it has several limitations that are addressed by AXIOME3. First, QIIME2 data visualization files (.qzv) requires the QIIME2 viewer, which adds an extra layer of complexity to end user biologists. Instead, AXIOME3 implements analysis and visualization within the same front-end interface, which simplifies the process for users. Second, QIIME2 Studio requires that users manually assemble individual workflow components. This limitation is addressed by AXIOME3 by enabling an automated workflow in which individual components are chained together into a single pipeline. This increased automation benefits users by simplifying repetitive workflows. The AXIOME3 pipeline also enables iterative data visualization and analysis, which allows users to easily interact with their data while customizing

and optimizing data visualizations. Importantly, AXIOME3 is not intended as a replacement of QIIME2 and QIIME2 Studio but is rather an automation tool that extends QIIME2 capabilities.

Availability of AXIOME3

AXIOME3 is an actively maintained and developed open-source project, and is available from GitHub (https://github.com/neufeld/AXIOME3; https://github.com/neufeld/AXIOME3-GUI). Note that only AXIOME3 GUI (https://github.com/neufeld/AXIOME3-GUI) needs to be installed for end users and doing so will automatically install the pipeline as well. The AXIOME3 pipeline repository (https://github.com/neufeld/AXIOME3) is exclusively intended for developers and collaborators. AXIOME3 is cross-platform compatible and the web GUI currently supports Chrome, Firefox, and Edge. The only other software requirement for AXIOME3 is Docker and Docker Compose, which is required to ensure a consistent build environment. A tutorial and sample dataset, as well as instructions about collaboration, are included in the AXIOME3 GUI project homepage.

Availability and Requirements

Project name: AXIOME3

Project home page: https://github.com/neufeld/AXIOME3 (AXIOME3 pipeline), https://github.com/neufeld/AXIOME3-GUI (AXIOME3 GUI)

Operating system(s): Platform independent

Browser support: Chrome, Firefox, Edge (AXIOME3 GUI)

Programming language: Python, Javascript

Other requirements: Docker (1.13.0+), Docker Compose (Version 3+)

License: BSD 3-Clause

Any restrictions to use by non-academics: No

Availability of Supporting Data

Snapshots of our code and other data further supporting this work are openly available in the GigaScience repository, GigaDB [13].

Abbreviations

SSU: small subunit; rRNA: ribosomal RNA; ASV: amplicon sequence variant; PCoA: principal coordinate analysis; OTU: operational taxonomic unit

Competing Interests

The authors declare no competing interests

Authors' contributions

DM designed and implemented AXIOME3 and prepared the manuscript. ACD and JDN contributed to design and coordination of AXIOME3 and manuscript preparation. All authors read and approved the final manuscript.

Acknowledgements

We thank Rachel Beaver, Katja Engel, Michelle McKnight, Emilie Spasov, Jackson Tsuji, and Alex Umbach for testing and suggestions during development. In addition to Discovery grants to ACD and JDN from the Natural Sciences and Engineering Research Council of Canada (NSERC), this research was supported by an Ontario Research Fund: Research Excellence (ORF-RE) grant and a Collaborative Research and Development (CRD) grant from NSERC, both in partnership with the Nuclear Waste Management Organization (NWMO).

References

[1] Bolyen E, Rideout JR, Dillon MR, Bokulich NA, Abnet CC, Al-Ghalith GA, et al. **Reproducible, interactive, scalable and extensible microbiome data science using QIIME 2**. *Nat. Biotechnol*. 2019; **37**:852–857.

[2] Schloss PD, Westcott SL, Ryabin T, Hall JR, Hartmann M, Hollister EB, et al. **Introducing mothur: Open-source, platform-independent, community-supported software for describing and comparing microbial communities.** *Appl Environ Microbiol*. 2009; **75:**7537-7541.

[3] Cole JR, Wang Q, Fish JA, Chai B, McGarrell DM, Sun Y, et al. **Ribosomal Database Project: Data and tools for high throughput rRNA analysis.** *Nucleic Acids Res*. 2014; **42:**D633–D642.

[4] Kim OS, Cho YJ, Lee K, Yoon SH, Kim M, Na H, et al. **Introducing EzTaxon-e: A prokaryotic 16S rRNA gene sequence database with phylotypes that represent uncultured species.** *Int J Syst Evol Microbiol*. 2012; **62:**716-721.

[5] Lynch MDJ, Masella AP, Hall MW, Bartram AK, Neufeld JD. **AXIOME: Automated exploration of microbial diversity.** *Gigascience*. 2013; **2:**3.

[6] Caporaso JG, Kuczynski J, Stombaugh J, Bittinger K, Bushman FD, Costello EK, et al. **QIIME allows analysis of high-throughput community sequencing data.** *Nat. Methods*. 2010; **7:**335–336.

[7] Carvalho BS, Rustici G. **The challenges of delivering bioinformatics training in the analysis of high-throughput data.** *Brief Bioinform*. 2013; **14:**538-547.

[8] Merkel D. **Docker: lightweight Linux containers for consistent development and deployment.** *Linux J*. 2014; **2014:**2.

[9] Callahan BJ, McMurdie PJ, Rosen MJ, Han AW, Johnson AJA, Holmes SP. DADA2: **High-resolution sample inference from Illumina amplicon data**. *Nat Methods*. 2016; **13:** 581–583.

[10] Quast C, Pruesse E, Yilmaz P, Gerken J, Schweer T, Yarza P, et al. **The SILVA ribosomal RNA gene database project: Improved data processing and web-based tools.** *Nucleic Acids Res*. 2013; **41:**D590-D596.

[11] Legendre P, Legendre L. **Chapter 9 - Ordination in reduced space.** In: Legendre P, Legendre L, editors. *Numer Ecol.* Amsterdam: Elsevier; 2012. p. 425–520.

[12] Borcard D, Gillet F, Legendre P, Borcard D, Gillet F, Legendre P. **Unconstrained Ordination.** In: *Numer Ecol with R. Use R*. Berlin: Springer; 2011. p. 115-151.

[13] Min D; Doxey AC; Neufeld JD: Supporting data for "AXIOME3:Automation, eXtension, and Integration Of Microbial Ecology" GigaScience Database. 2021. http://dx.doi.org/10.5524/100854.

Figure legends

Figure 1. Schematic representation of the AXIOME3 pipeline workflow.

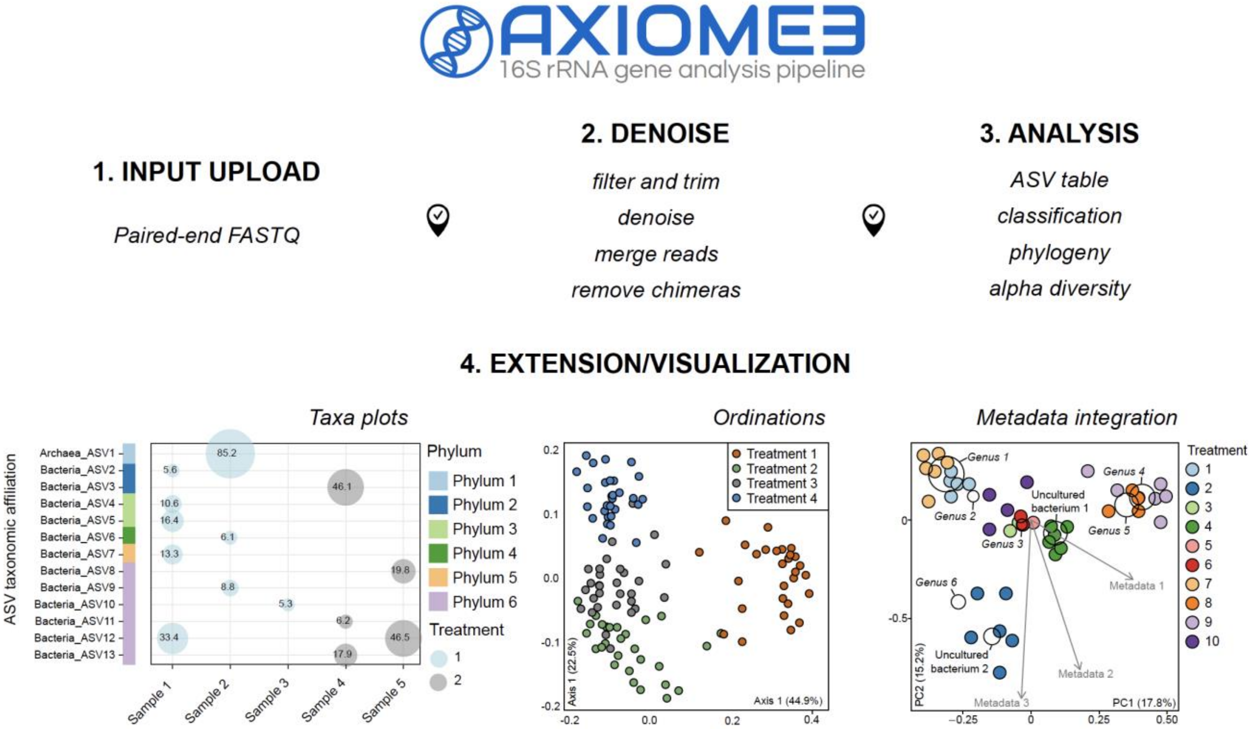

Click here to access/download;Figure;AXIOME3_Figure_1.pdf

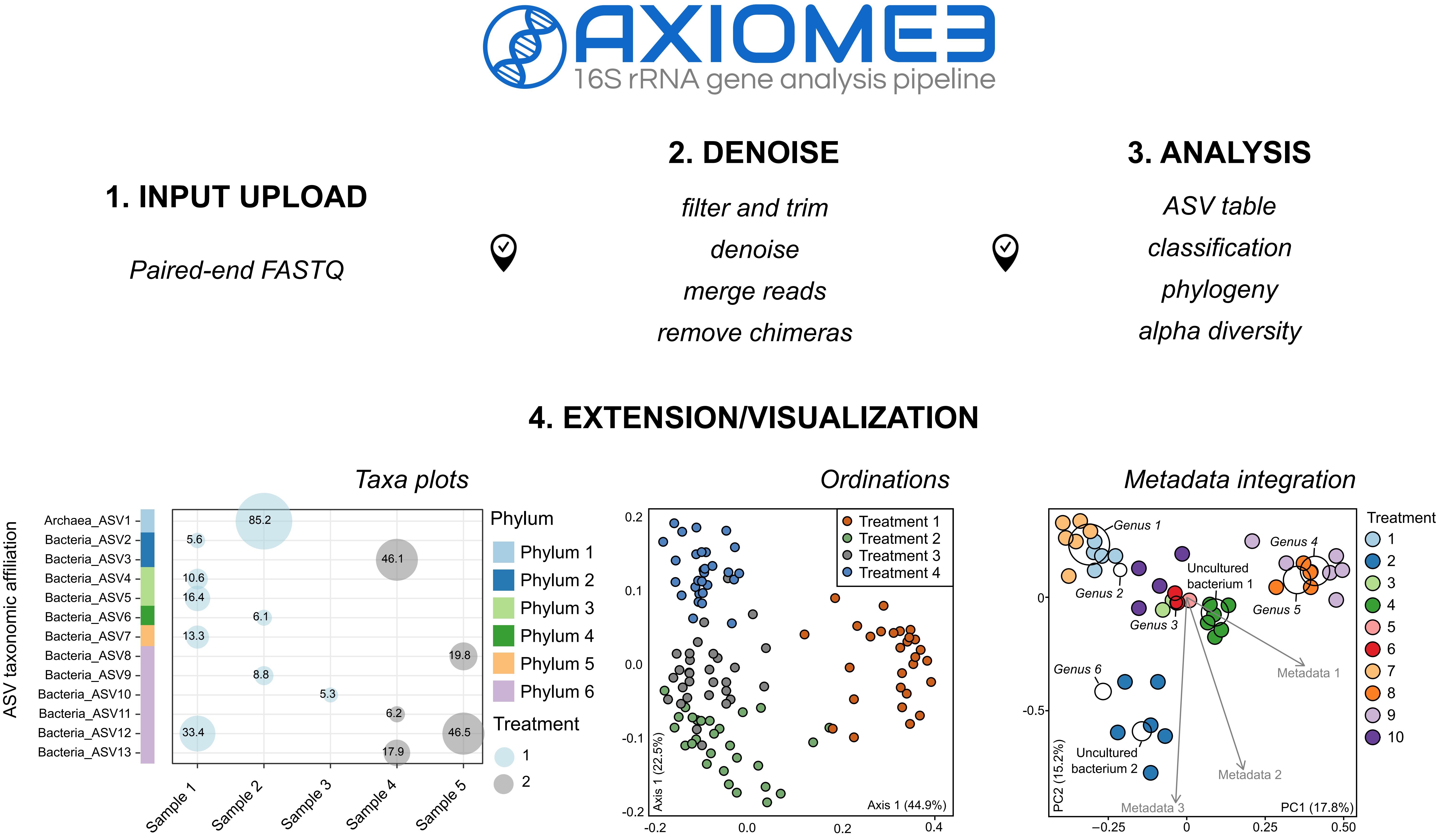

Coverletter
Click here to access/download;Personal Cover;AXIOME3_coverletter.pdf

UNIVERSITY OF WATERLOO

DEPARTMENT OF BIOLOGY
519-888-4567 ext. 32569 | fax 519-746-0614
uwaterloo.ca/biology

GigaScience; November 24, 2020

Dear Editor,

We hereby submit a revised manuscript entitled “AXIOME3: Automation, eXtension, and Integration Of Microbial Ecology” for publication as an Technical Note in GigaScience. The software has not been submitted for publication elsewhere while under consideration for GigaScience. A single figure is included at the end of the upload file.

Thank you for soliciting such a rapid review process with expert referees who used our software and provided valuable suggestions for increasing its usability. We’ve addressed all comments and have updated code, websites, and files provided to users. AXIOME3 and the associated manuscript is now much improved.

Thanks again for guiding this process and we hope that you will now find our manuscript suitable for publication in GigaScience.

Sincerely,

Josh D. Neufeld; Professor

Rebuttal Click here to access/download;Personal Cover;AXIOME3_rebuttal.pdf

REVIEWER 1

The authors present Axiome3, essentially a GUI pipeline wrapper for 1) Qiime2 scripts containing 2) a novel extension module enabling generation of common figures and analyses used in microbial ecology.

Overall, I see Axiome3 as a relevant addition to the rRNA gene amplicons bioinformatics pipeline landscape. My main concern however is that the authors are pushing the scope of their pipeline too far in the manuscript where they often refer to their method as being a solution for rRNA amplicon data analysis that should be used for large scale and repeated analyses. Below I elaborate with more details on why I disagree with these claims.

Although we very much think that AXIOME3 can be a solution for large scale and repeated analyses, we are nonetheless willing to revise manuscript wording around this to satisfy the reviewer concern, as specified below.

That said, I definitely see potential in axiome3 : Qiime2 (even for an experienced bioinformatician) can be a pain to use, especially the initial steps were one has to setup cryptic metadata files that requires lots of attempts to make it work. In that regard, axiome3 could prove to be objectively useful in providing a robust GUI-wrapper for Qiime2. I think the authors should modify the tone of the manuscript to make it explicit that axiome3 targets microbiologists end users unfamiliar with Linux/command line/ HPC environments and who have a need to analyze a « normal » dataset in a timely manner - which is totally fine. By experience large scale (and more complex) data analysis will pretty much always be performed on a Linux HPC environment (faster, easier to debug and more flexible).

We agree with the reviewer suggestion to modify the tone. Our revised manuscript now indicates that AXIOME3 is particularly suited for use by individual labs with a wide range of users, where many may be unfamiliar with a Linux operating system and command line data manipulations.

Extension module :
The extension module part looks interesting. I suggest the authors put on some instructions on how someone could add a module/functionality.

We agree and have added instructions about collaboration on the project homepage.

Specific comments :

Lines 14-15 : « not be suitable for fast-paced and large-scale data analysis by a wide range of end user microbiologists » This is highly debatable : one might argue that both fast-paced and large-scale are simply not prone to being analyzed by an untrained computational biologist. It is true however that these analyses may not be prone to be executed by end user microbiologists unfamiliar with a command line environment. Please modify accordingly.

We have revised the text to read, “However, existing tools often require programming ability and frequent user intervention that may not be suitable for fast-paced and large-scale data analysis by end user microbiologists who are unfamiliar with the Linux command line environment or prefer interactions with a graphical user interface.”

Line 28 and on : « Because mass collection of SSU rRNA gene amplicon sequence data presents a challenge to efficiently analyze the large-scale data in a timely manner» I mostly disagree with that statement. In the grand scheme of things, rRNA gene amplicon data processing is not that complex and does not require important computing resources (say compared to shotgun metagenomics or eukaryote genomes assembly and many other fields relying on computing).

We agree that metagenomic or eukaryotic genome analyses require much more computing resources and time compared to amplicon data. Instead of focusing on the scale of the data, we have reworded this statement to emphasize instead that repeat analysis of amplicon sequence data can be labour intensive in a command line environment.

And then « AXIOME3 now offers rapid and user-friendly options within an automated pipeline, with advanced data visualization tools and the ...» More realistically, the large scale analysis of thousands of rRNA gene amplicon data will likely be done by a computational biologist or bioinformatician who is comfortable in a linux HPC environment. In the context of Axiome3, it would be more appropriate to promote the package as a solution for microbiologist untrained at the command line that wants to analyze their own data (likely less than a thousand samples for a « standard » rRNA amplicon profiling project) for which there is certainly a need in the community.

As indicated above, we agree and have updated the text to emphasize that AXIOME3 is helpful for repetitive amplicon sequence data analysis by microbiologists who are less familiar with command line environments or who prefer GUI interactions.

Line 41 : « which may limit the efficiency of frequent repetitive analyses »

I again disagree : Qiime2, mothur, etc have all their pros and cons, but they are certainly okay for repetitive analyses because they are command line-based. A GUI, by its inherent nature, is not prone for frequent repetitive analyses.

Although command line tools can technically be used for repetitive analyses, researchers would still have to run individual workflow components with extensive user intervention, which can become tedious and relatively labour intensive. Automating the analysis pipeline, as AXIOME3 does, greatly reduces workloads for researchers in our labs. Because a GUI is not ideally suited for frequent repetitive analyses, AXIOME3 comes with both the pipeline and the GUI. It is the pipeline portion that is intended for frequent repetitive analyses, not the GUI portion. We have updated the manuscript to clarify this distinction and prevent confusion by future readers.

Line 72 : please replace with some along the lines of : ...to makes research more accessible…

We have corrected this in the manuscript as suggested.

Line 85 : « Because several amplicon data analysis tasks are time consuming and resource heavy » Again I think this statement is mileading. Strickly speaking, 16S rRNA amplicon data processing is not resource heavy and not time consuming compared to other bioinformatics workflow. Please remove.

We agree and have removed this statement.

Installation and testing :
I managed to properly install axiome and axiome-gui. The instructions were clear. However, maybe I missed it, but I did not see any detailed instructions on how to link the GUI to a server or compute cluster. Is it possible to install the pipeline on a server and run the pipeline from the user's local GUI? If so detailed instructions on how to do this would be a great addition.

Yes, this is possible by accessing the server with SSH and we have updated the corresponding instructions on the project homepage.

In both the [Input upload] and [denoise] tabs, I could not load a simple manifest file pointing to a dummy fastq dataset. It returned an error msg in a window : « Manifest file headers are not compatible with QIIME2 manifest format! ». Although I'm quite certain I generated my manifest files according to the instructions, maybe I did something wrong. But since these files are important for the pipeline's execution, I suggest the authors to

make a test dataset available with their pipeline (maybe a public repository like zenodo.org? Or directly in the pipeline repository?). More specifically please make a valid (and tested) manifest file available with the test data.

Agreed. We have updated the AXIOME3 usage guide and included the test dataset and tutorials.

I look forward to see these test datasets so I can actually test Axiome3.

The test dataset and tutorials are now available.

REVIEWER 2

Summary: AXIOME3 SSU rRNA analysis pipeline that combines QIIME2, R, and Python into a docker container which makes it easy to download, install, and run and enhances/improves ease of use of QIIME2. The web graphical user interface allows for user-friendly QIIME2 analysis and data visualizations of results. AXIOME3 is the 3rd version of the AXIOME software package, which has been well maintained, developed, and documented. Three areas of potential improvement: (i) a dockerless AXIOME3 web interface, (ii) more detailed status and error messages while running the software, (iii) more flexibility with respect to user options and customizations. Overall, the software is very useful and the manuscript was well-written, but a few issues made it not as user-friendly as advertised.

(i) a dockerless AXIOME3 web interface

Docker makes software installation much easier than manual installation because Docker will take care of all the package and operating system dependencies for users. Another option is to package AXIOME3 with a software installer, which prompts users to follow a series of installation steps. However, as much as we would like to make a Dockerless application option, this would not be possible to achieve in a short period of time because it would require changing the entire software architecture. Although installing Docker may be challenging for some users without much software experience, Docker installation needs to be done only once. The goal of AXIOME3 (and using Docker) was to simplify the experience for users; moving toward a Docker-less installation would be inconsistent with this goal.

(ii) more detailed status and error messages while running the software

We agree with this suggestion and have updated status and error messages for AXIOME3 users.

(iii) more flexibility with respect to user options and customizations

We have made several updates to user options and customizations in response to this suggestion.

Major revisions
1) "Comparison to Related Work" section compares to QIIME2, but doesn't discuss how AXIOME3 compares to AXIOME and AXIOME2.

We agree and have added a section that describes how AXIOME3 is different from AXIOME. Although somewhat awkward, AXIOME2 was never published, yet was functionally identical to AXIOME. We now describe this in the text and mention AXIOME2 (unpublished) specifically.

a) Given this is the 3rd version in a successful line of tools for microbiome analyses, a table is needed that compares/contrasts AXOIME3 to the previous versions with respect to features and functionality, especially given it is a complete redesign/refresh.

Instead of a table, we have described the distinct functionality of AXIOME3, which is predominantly associated with QIIME2 compatibility. Although we will defer to the editor's preference, we would rather avoid creating a table that can instead be communicated with a sentence or two.

2) One of the strong selling points of the software is that it is easier to use than QIIME2. However, improvements can still be made to enhance AXIOME3's ease-of-use:
a) First, AXIOME requires five steps to finish the whole analysis (from denoise to taxonomic classification), which is roughly the same amount of steps QIIME2 requires.

We should clarify that AXIOME3 does not require five steps. Rather, it has three (upload, denoise, and analysis). In addition, QIIME2 can require many more steps depending on what researchers wish to achieve. For example, if all AXIOME3 analyses were conducted with a native QIIME2 install, there would be many more steps that include upload, denoise, taxonomic classification, phylogeny tree, diversity metrics, and multiple data visualizations and exports. Each would require running individual

commands for each export and visualization. In response to this comment we have made some minor manuscript edits to clarify.

b) Second, it lacks error messages at times. Whenever AXIOME3 encounters a bug, it doesn't always show an error message. On the other hand, QIIME2 always displays some error messages to guide users when debugging.

AXIOME3 has two main sources of errors: general software related errors and QIIME2 related errors. We agree that general software related errors were somewhat difficult to understand for users and we have made improvements in response to this concern. However, we should clarify that AXIOME3 always directly relays QIIME2 error messages back to users, so users have some debugging information if the failures are QIIME2 related. Thus, we have improved error handling for general AXIOME3 software errors but have not made any changes to QIIME2-specific errors.

c) Third, in order to automate QIIME2 pipeline, AXIOME3 offers reduced flexibility. Specifically, users can only choose DADA2 for denoising and a classifier trained on the SILVA database.

Currently, QIIME2 offers two options for denoising: DADA2 and Deblur. We did not include the Deblur plugin because it does not support paired-end reads. In the future, we plan to add more denoising options such as Deblur (once its QIIME2 plugin supports paired-end reads) and PANDAseq. However, the vast majority of users currently use DADA2 so this remains a low priority change.

There is no restriction on the type of database that can be used to train the classifier as long as it is trained using QIIME2. However, because different databases will have unique naming conventions, this may cause an error because the bubble plot and triplot tools in the Extension module assume that the taxonomic names follow the SILVA database naming convention. In response to this concern, we have tested RDP classification options and have updated the manuscript to mention this as an additional database that users can use.

d) Fourth, while the web graphical user interface is a very nice addition, it requires users to install docker, which might create obstacles for those with little to no computational background. It would be much easier if users can use it without installing anything else, such as a web based application.

As described above, we will maintain a Docker installation because of our design philosophy (ease of use) and existing software architecture. Nonetheless, we have

included Docker installation advice on our project homepage to help users who may find a Docker installation difficult.

Software evaluation
1) Download and install
a) Successfully installed GUI (https://github.com/neufeld/AXIOME3-GUI)
b) Ran into resolvePackageNotFound with pipeline (https://github.com/neufeld/AXIOME3).

We should clarify that users are not supposed to install the pipeline itself. When users install AXIOME3-GUI, as described in the instructions on the project homepage, AXIOME3 pipeline will automatically be installed inside the Docker container. We have clarified this in the manuscript for future readers.

2) Running step-by-step:
a) Step 1: input upload: Spent ~45min until receiving a viable manifest file. When trying .tsv files, continually received an error:"Manifest file headers are not compatible with QIIME2 manifest format!" Only success was with the manifest file for "PairedEndFastqManifestPhred33" formatted .txt file.

The example Manifest file in AXIOME3 was accidentally in the wrong format. We have fixed the file and have included a test dataset and sample manifest file on the project homepage for users convenience.

b) Step 2: denoise: successful and clear documentation. However, link for download denoise summary (.qzv) instead downloaded feature table (.qza)

We thank the reviewer for finding this bug - the error has been corrected.

c) Step 3: analysis: Task failed due to no space left on device. This was a user error and AXIOME3 caught it.

This is helpful feedback - thank you.

d) Finally for the viewer, "Upload the "QIIME2 Visualization (.qzv)" file in the QIIME2 Viewer". A bit surprised the user is expected to also use QIIME2 Viewer while using AXIOME3.

In AXIOME3, the QIIME2 Viewer is used once to visualize the input sequence quality because it is difficult to display such data without substantial loss of information. The

QIIME2 Viewer offers an excellent interactive graph for this particular use case. All other visualizations are embedded in AXIOME3 because they can be displayed without loss of information. Also, AXIOME3 is not meant to replace QIIME2 and its associated software. Users may use QIIME2 and the associated software in addition to AXIOME3 whenever necessary.

e) README is clear and contains detailed instructions. Great visuals to assist with the process.

Good feedback - thank you.

f) We did not find any complete examples or tutorials for users to run or test. It would have been helpful to have an example with a manifest file and sample sheet to the users instead of the pseudo manifest file in the README.

Apologies - the tutorial and test dataset are now available on the project homepage.
