# Supplementary material for: AXIOME3: Automation, eXtension, and Integration Of Microbial Ecology
Source: Gigascience. 2021 Feb 3;10(2):giab006. doi: 10.1093/gigascience/giab006 (PMC7931817; doi:10.1093/gigascience/giab006)
Supplement: giab006_Response_to_Reviewer_Comments_Original_Submission [file giab006_response_to_reviewer_comments_original_submission.pdf]

# Author's Response To Reviewer Comments

Close

See attached file (with italics used to distinguish reviewer comments)

Close
