# Supplementary material for: AXIOME3: Automation, eXtension, and Integration Of Microbial Ecology
Source: Gigascience. 2021 Feb 3;10(2):giab006. doi: 10.1093/gigascience/giab006 (PMC7931817; doi:10.1093/gigascience/giab006)
Supplement: giab006_Reviewer_1_Report_Original_Submission — Julien Tremblay -- 10/14/2020 Reviewed [file giab006_reviewer_1_report_original_submission.pdf]

Reviewer Report

Title: AXIOME3: Automation, eXtension, and Integration Of Microbial Ecology

Version: Original Submission **Date:** 10/14/2020

Reviewer name: Julien Tremblay

Reviewer Comments to Author:

Main comments :
The authors present Axiome3, essentially a GUI pipeline wrapper for 1) Qiime2 scripts containing 2) a novel extension module enabling generation of common figures and analyses used in microbial ecology. Overall, I see Axiome3 as a relevant addition to the rRNA gene amplicons bioinformatics pipeline landscape. My main concern however is that the authors are pushing the scope of their pipeline too far in the manuscript where they often refer their method as being a solution for rRNA amplicon data analysis that should be used for large scale and repeated analyses. Below I elaborate with more details on why I disagree with these claims.
That said, I definitely see potential in axiome3 : Qiime2 (even for an experienced bioinformatician) can be a pain to use, especially the initial steps were one has to setup cryptic metadata files that requires lots of attempts to make it work. In that regard, axiome3 could prove to be objectively useful in providing a robust GUI-wrapper for Qiime2. I think the authors should modify the tone of the manuscript to make it explicit that axiome3 targets microbiologists end users unfamiliar with Linux/command line/ HPC environments and who have a need to analyze a Â« normal Â» dataset in a timely manner - which is totally fine. By experience large scale (and more complex) data analysis will pretty much always be performed on a Linux HPC environment (faster, easier to debug and more flexible).
Extension module :
The extension module part looks interesting. I suggest the authors put on some instructions on how someone could add a module/functionality.
Specific comments :
Lines 14-15 : Â« not be suitable for fast-paced and large-scale data analysis by a wide range of end user microbiologists Â»
This is highly debatable : one might argue that both fast-paced and large-scale are simply not prone to being analyzed by an untrained computational biologist. It is true however that these analyses may not be prone to be executed by end user microbiologists unfamiliar with a command line environment. Please modify accordingly.
Line 28 and on : Â« Because mass collection of SSU rRNA gene amplicon sequence data presents a challenge to efficiently analyze the large-scale data in a timely manner Â»
I mostly disagree with that statement. In the grand scheme of things, rRNA gene amplicon data processing is not that complex and does not require important computing resources (say compared to shotgun metagenomics or eukaryote genomes assembly and many other fields relying on computing). And then Â« AXIOME3 now offers rapid and user-friendly options within an automated pipeline, with

advanced data visualization tools and the ...Â»
More realistically, the large scale analysis of thousands of rRNA gene amplicon data will likely be done by a computational biologist or bioinformatician who is comfortable in a linux HPC environment. In the context of Axiome3, it would be more appropriate to promote the package as a solution for microbiologist untrained at the command line that wants to analyze their own data (likely less than a thousand samples for a Â« standard Â» rRNA amplicon profiling project) for which there is certainly a need in the community.
Line 41 : Â« which may limit the efficiency of frequent repetitive analyses Â»
I again disagree : Qiime2, mothur, etc have all their pros and cons, but they are certainly okay for repetivive analyses because they are command line-based. A GUI, by its inherent nature, is not prone for frequent repetitive analyses.
Line 72 : please replace with some along the lines of : ...to makes research more accessible…
Line 85 : Â« Because several amplicon data analysis tasks are time consuming and resource heavy Â»
Again I think this statement is mileading. Strickly speaking, 16S rRNA amplicon data processing is not resource heavy and not time consuming compared to other bioinformatics workflow. Please remove.
Installation and testing :
I managed to properly install axiome and axiome-gui. The instructions were clear. However, maybe I missed it, but I did not see any detailed instructions on how to link the GUI to a server or compute cluster. Is it possible to install the pipeline on a server and run the pipeline from the user's local GUI? If so detailed instructions on how to do this would be a great addition.
In both the [Input upload] and [denoise] tabs, I could not load a simple manifest file pointing to a dummy fastq dataset. It returned an error msg in a window : Â« Manifest file headers are not compatible with QIIME2 manifest format! Â». Although I'm quite certain I generated my manifest files according to the instructions, maybe I did something wrong. But since these files are important for the pipeline's execution, I suggest the authors to make a test dataset available with their pipeline (maybe a public repository like zenodo.org? Or directly in the pipeline repository?). More specifically please make a valid (and tested) manifest file available with the test data.
I look forward to see these test datasets so I can actually test Axiome3.

Level of Interest

Please indicate how interesting you found the manuscript: Choose an item.

Quality of Written English

Please indicate the quality of language in the manuscript: Choose an item.

Declaration of Competing Interests

Please complete a declaration of competing interests, considering the following questions:

- Have you in the past five years received reimbursements, fees, funding, or salary from an organisation that may in any way gain or lose financially from the publication of this manuscript, either now or in the future?
- Do you hold any stocks or shares in an organisation that may in any way gain or lose financially from the publication of this manuscript, either now or in the future?
- Do you hold or are you currently applying for any patents relating to the content of the manuscript?
- Have you received reimbursements, fees, funding, or salary from an organization that holds or has applied for patents relating to the content of the manuscript?
- Do you have any other financial competing interests?
- Do you have any non-financial competing interests in relation to this paper?

If you can answer no to all of the above, write 'I declare that I have no competing interests' below. If your reply is yes to any, please give details below.

I declare that I have no competing interests

I agree to the open peer review policy of the journal. I understand that my name will be included on my report to the authors and, if the manuscript is accepted for publication, my named report including any attachments I upload will be posted on the website along with the authors' responses. I agree for my report to be made available under an Open Access Creative Commons CC-BY license (http://creativecommons.org/licenses/by/4.0/). I understand that any comments which I do not wish to be included in my named report can be included as confidential comments to the editors, which will not be published.

Choose an item.

To further support our reviewers, we have joined with Publons, where you can gain additional credit to further highlight your hard work (see: https://publons.com/journal/530/gigascience). On publication of this paper, your review will be automatically added to Publons, you can then choose whether or not to claim your Publons credit. I understand this statement.

Yes Choose an item.
